# Overexpression of 17β-hydroxysteroid dehydrogenase type 10 increases pheochromocytoma cell growth and resistance to cell death

**DOI:** 10.1186/s12885-015-1173-5

**Published:** 2015-03-22

**Authors:** Emily A Carlson, Rebecca T Marquez, Fang Du, Yongfu Wang, Liang Xu, Shirley ShiDu Yan

**Affiliations:** 1 Department of Pharmacology & Toxicology, University of Kansas, Lawrence, KS 66047 USA; 2 Higuchi Biosciences Center, University of Kansas, Lawrence, KS 66047 USA; 3 Department of Molecular Biosciences, University of Kansas, Lawrence, KS 66047 USA

**Keywords:** 17β-hydroxysteroid dehydrogenase type 10, Mitochondrial alteration, Pheochromocytoma, Cancer development

## Abstract

**Background:**

17β-hydroxysteroid dehydrogenase type 10 (HSD10) has been shown to play a protective role in cells undergoing stress. Upregulation of HSD10 under nutrient-limiting conditions leads to recovery of a homeostatic state. Across disease states, increased HSD10 levels can have a profound and varied impact, such as beneficial in Parkinson’s disease and harmful in Alzheimer’s disease. Recently, HSD10 overexpression has been observed in some prostate and bone cancers, consistently correlating with poor patient prognosis. As the role of HSD10 in cancer remains underexplored, we propose that cancer cells utilize this enzyme to promote cancer cell survival under cell death conditions.

**Methods:**

The proliferative effect of HSD10 was examined in transfected pheochromocytoma cells by growth curve analysis and a xenograft model. Fluctuations in mitochondrial bioenergetics were evaluated by electron transport chain complex enzyme activity assays and energy production. Additionally, the effect of HSD10 on pheochromocytoma resistance to cell death was investigated using TUNEL staining, MTT, and complex IV enzyme activity assays.

**Results:**

In this study, we examined the tumor-promoting effect of HSD10 in pheochromocytoma cells. Overexpression of HSD10 increased pheochromocytoma cell growth in both *in vitro* cell culture and an *in vivo* xenograft mouse model. The increases in respiratory enzymes and energy generation observed in HSD10-overexpressing cells likely supported the accelerated growth rate observed. Furthermore, cells overexpressing HSD10 were more resistant to oxidative stress-induced perturbation.

**Conclusions:**

Our findings demonstrate that overexpression of HSD10 accelerates pheochromocytoma cell growth, enhances cell respiration, and increases cellular resistance to cell death induction. This suggests that blockade of HSD10 may halt and/or prevent cancer growth, thus providing a promising novel target for cancer patients as a screening or therapeutic option.

## Background

While early detection and improved treatments have helped manage certain types of cancer incidence and death rates [1], this vast group of diseases still accounts for approximately 15% of all deaths worldwide each year [2]. Rapid replication of abnormal cells and resistance to available anticancer treatments are two prominent factors underlying cancer aggressiveness [3]. As every patient’s cancer is distinctively different, defining the underlying mechanisms may implicate tumor-specific molecular agents. Identification of such players could lead to novel targets for tailored therapies unique to each individual.

The mechanisms underlying cancer have been widely studied, with much emphasis placed on mitochondria [4]. Since Otto Warburg first showed that tumor cells exhibit increased glycolytic adenosine triphosphate (ATP) production and reduced oxidative phosphorylation [5], the mitochondrion has undergone thorough investigation to elucidate the alterations promoting cancer. It is well known that constant reactive oxygen species (ROS) exposure induces mutations in mitochondrial DNA, which lead to cancer initiation and metastasis [6]. While elevated ROS levels cause substantial intracellular damage, tumor cells are capable of rebalancing ROS production and elimination by activating antioxidants [7]. By balancing ROS levels, tumor cells can undergo unhindered, continual proliferation. Moreover, cancer cells display elevated inhibition of mitochondrial-mediated cell death, regardless of ROS accumulation [8]. Modification of the mitochondrial permeability transition pore (MPTP) in malignant cells has been shown to render cells more resistant to anticancer therapies [9], however, the mechanism underlying this resistance is not fully understood.

HSD10 (17β-hydroxysteroid dehydrogenase type 10), also known as ABAD (amyloid-β-binding alcohol dehydrogenase) and MHBD (2-methyl-3-hydroxybutyryl-CoA dehydrogenase), is a mitochondrial enzyme that catalyzes the oxidation of a wide variety of alcohols, fatty acids, and steroids [10]. Our group originally identified HSD10 as a single chain, ≈27 kilodalton polypeptide capable of binding amyloid-β [11,12]. HSD10 is crucial for the maintenance of cellular homeostasis, and overexpression has been shown to provide a protective effect in cells undergoing nutritional stress [13]. HSD10 belongs to the short-chain dehydrogenase/reductase superfamily 17β-hydroxysteroid dehydrogenase (HSD17B). These alcohol oxidoreductases catalyze the dehydrogenation of 17-hydroxysteroids [14]. Several HSD17B family members have been implicated in breast [15-17], endometrial [18], and colorectal cancers [19], suggesting that this group of enzymes is important in different types of cancer. HSD10 was shown to be elevated in certain prostate carcinomas and osteosarcomas [20,21], indicative of a potential role in cancer. Furthermore, HSD10 may promote tumorigenesis and aggressiveness, as elevated HSD10 levels were observed in prostate-to-bone metastases compared to non-malignant prostate and primary prostate tumor tissue [22]. While HSD10 remains underexplored in all cancer types, the current data in bone and prostate cancers strongly suggest that HSD10 may be utilized in cancer cells for protection against cell death and enhancement of unrestricted growth.

Based on these observations, we postulate that HSD10 overexpression enhances cancer cell growth and resistance to cell death. To examine the tumorigenic capability of HSD10, we selected the rat adrenal gland (pheochromocytoma) tumor cell line, PC-12. Here, we provide evidence that HSD10 mediates pheochromocytoma cell growth in cell culture and in a mouse model. Furthermore, we have identified a novel HSD10 binding partner, Cyclophilin D (CypD), a regulatory component of the MPTP, which may provide increased resistance to cell death in tumor cells. Now that we have demonstrated the effect of overexpressing HSD10 in non-malignant tumor cells, our future efforts will focus on evaluating the role of HSD10 in multiple types of cancer.

## Methods

### Materials

Reagents were obtained from the following sources: RPMI-1640 medium, Hank’s balanced salt solution (HBSS), 0.25% Trypsin-EDTA, heat-inactivated horse serum, fetal bovine serum (FBS), G418 supplement, Lipofectamine 2000, Mito Tracker Red, Mito Tracker Green, tetramethylrhodamine methyl ester (TMRM) from Invitrogen Co. (Grand Island, NY); Bicinchoninic Acid (BCA) protein assay from Pierce Chemical Company (Rockford, IL); CellTiter 96 Non-Radioactive Cell Proliferation Assay from Promega Co. (Madison, WI); Lentiviral Packaging Mix, Tert-Butyl hydroperoxide (TBH), Coenzyme Q_1_, Coenzyme Q_2_ from Sigma-Aldrich Co. (St. Louis, MO); Hydrogen peroxide (H_2_O_2_), Super Signal West Pico Chemiluminescent Substrate from Thermo Fisher Scientific Co. (Waltham, MA); ATP Bioluminescence Assay Kit HS II, *In Situ* Cell Death Detection Kit, Fluorescein from Roche Applied Science Co. (Indianapolis, IN); Signal transduction antibodies from Cell Signaling Technology Co. (Danvers, MA). All other chemicals used were of the highest purity commercially available.

### Generation of stably transfected PC-12 cells overexpressing HSD10

The rat pheochromocytoma (adrenal gland tumor) cell line PC-12 (ATCC® CRL-1721, Manassas, VA) was used for stable transfection of HSD10 as formerly described [13]. In brief, PC-12 cells (10^5^ cells) were transfected with pcDNA3/(human) wild-type HSD10, or pcDNA3 alone (vector) previously linearized with *Sma*I, using Lipofectamine 2000 [23]. 48 hours after transfection, cells were plated at 1:10–1:20 dilution in 100-mm dishes supplemented with 1 mg/ml G418. After 1–2 weeks, single clones were isolated, and cells were separated with trypsin, subjected to limiting dilution, and replated in medium containing 1 mg/ml G418 for 2–4 weeks. After, cells were maintained in RPMI-1640 medium supplemented with 10% horse serum, 5% FBS, and 1 mg/ml G418 in a humidified 37°C, 5% CO_2_ environment.

### Generation of lentiviral transfected PC-12 cells under-expressing HSD10

HEK 293 T cells (ATCC® CRL-11268; 2.5 × 10^5^ cells/well in a 6-well plate) were transfected with HSD10 shRNA (HSD17B10 MISSION® shRNA Bacterial Glycerol Stock, Human, SHCLNG NM_004493.2-751s21c1 TRCN0000318938, Sequence: CCGGCATCGAGAACCCATTCCTCAACTCGAGTTGAGGAATGGGTTCTCGATGTTTTTG; Sigma-Aldrich) or control shRNA (MISSION® TRC2 pLKO.5-puro Non-Mammalian shRNA Control Plasmid DNA, SHC202; Sigma-Aldrich) using Lipofectamine 2000 and Lentiviral Packaging Mix. Cell media was harvested at 36 and 72 hours post-transfection. PC-12 cells (10^5^ cells/well in a 12-well plate) were infected with HSD10 shRNA and non-mammalian shRNA media over 10 days with continual passaging, and then were maintained in RPMI-1640 medium supplemented with 10% horse serum and 5% FBS in a humidified 37°C, 5% CO_2_ incubator, with shRNA viral media added to cell cultures once a week.

### Characterization of transfected PC-12 cell lines

All PC-12 cell lines, between passages 1–8, were characterized using immunoblotting to determine HSD10 expression level, immunofluorescence staining to verify HSD10 expression level and localization, and growth curves.

#### Immunoblotting

Whole cell lysates were prepared from harvested cells. Cells were washed twice with pre-chilled phosphate buffered saline (PBS), followed by detachment with a scraper and sonication. Lysates were measured for protein concentration using the BCA protein assay. Proteins from the lysates were separated on NuPAGE® Novex® Bis-Tris 10% and 12% gels (Invitrogen) by SDS-PAGE. Gels were transferred to 0.45 μm nitrocellulose membranes (Thermo Fisher Scientific) and probed with rabbit anti-HSD10 IgG (generated in our laboratory [11], 1:3000) and mouse anti-Actin (1:8000). HSD10 was visualized using a chemiluminescence mixture and FluorChem HD2 imaging system (ProteinSimple, Santa Clara, CA).

#### Immunofluorescence staining

Cells (2 × 10^4^ cells/well) were grown in 8-well chamber slides until 70% confluent, and then incubated with 100 nM Mito Tracker Red for 30 minutes, followed by fixation in 4% paraformaldehyde and 0.1% Triton X-100 for 30 minutes. Fixed cells were incubated with rabbit anti-HSD10 IgG (1:200, generated in our laboratory) at 4°C overnight. The same concentration of non-immune IgG was used as a control. Secondary antibody (Alexa Fluor 488 anti-rabbit IgG, 1:2000, Invitrogen) was applied to the cells followed by confocal microscopy (Leica Microsystems, Buffalo Grove, IL). The intensity of fluorescence (ex: 581 nm, em: 644 nm for Mito Tracker Red; ex: 499 nm, em: 520 nm for HSD10) was recorded to determine HSD10 expression and mitochondrial localization.

#### Growth curves

Cells were plated in 100-mm dishes at a density of 2.5 × 10^5^ cells/dish. One dish was chosen each consecutive day to be counted. The cells in the dish were detached with Trypsin, centrifuged, and resuspended in 1 ml of DMEM media, followed by counting using a hemocytometer.

### Mitochondrial function and cell death assays

Mitochondrial membrane potential and bioenergetics were determined as previously described [24,25] in the PC-12 altered cell lines at passages 1–8.

#### Fluorescence staining of TMRM to examine mitochondrial membrane potential

Cells (2 × 10^4^ cells/well) were grown in 8-well chamber slides until 70% confluent, and then incubated with 150 nM Mito Tracker Green (Invitrogen) and 100 nM TMRM (non-quench mode) for 30 minutes, followed by washing twice with HBSS media. Cells were imaged live by confocal microscopy and the intensity of fluorescence (ex: 490 nm, em: 516 nm for Mito Tracker Green; ex: 490 nm, em: 550 nm for TMRM) was recorded to determine the uncollapsed proton gradient.

#### Measurement of cellular ATP

Cellular ATP levels were measured using Bioluminescence Assay Kit HS II following the manufacturer’s instructions as we previously described [25]. Briefly, cells (30 × 10^4^ cells/well) were grown in 6-well plates until fully confluent. Cells were washed twice with ice-cold PBS, followed by the addition of 50 μl/well of ATP Lysis Buffer. Cells were harvested, incubated on ice for 20 minutes, and then centrifuged. Protein content of the supernatant was determined using the BCA protein assay. Proportional amounts of sample were added to a 96-well ATP plate (25 μl/well). The reaction solution was brought to 50 μl/well with the addition of ATP Dilution Buffer. ATP levels were determined using an LMax II 384 Microplate Reader and SoftMax Pro (Molecular Devices, Sunnyvale, CA).

#### Measurement of cellular metabolic activity

The CellTiter 96 Non-Radioactive Cell Proliferation Assay kit was used to measure metabolic activity. Cells (10^4^ cells/well) were plated in 96-well plates 48 hours prior to the experiment. For cellular resistance experiments, H_2_O_2_ (0.1, 0.25, 0.5, 0.75, and 1 mM) and TBH (0.1 and 0.25 mM) were added to cells 24 hours prior to incubation with 15 μl/well of MTT (3-(4,5-dimethylthiazol-2-yl)-2,5-diphenyltetrazolium bromide) dye solution for 4 hours. Successively, 100 μl/well of solubilization solution was added to stop the reaction, and then the plates were incubated for 1 hour. The change in absorbance at 570 nm was recorded using a 96-well Synergy HT Multi-Mode Microplate Reader (BioTek Instruments, Winooski, VT). A reference wavelength of 650 nm was taken to reduce background.

#### Measurement of enzyme activities associated with respiratory chain complexes

Briefly, cells (10^6^ cells/dish) were grown in 150-mm dishes until fully confluent. For measurement of complex I-IV enzymatic activities in response to oxidative stress, 0.75 mM of H_2_O_2_ was added to cells and incubated for 1, 6, 16, and 24 hours before collection. Cells were washed twice with pre-chilled PBS, and then harvested, centrifuged, and suspended in 100 μl of isolation buffer containing 225 mM D-mannitol, 75 mM sucrose, and 2 mM K_2_HPO_4_. A working solution of 25 mM potassium (K) buffer (3 M KCl, 1 M Tris–HCl, pH 7.4, and 0.5 M EDTA) was used for complex I, II, III, and IV assays as described [25]. An Ultrospect 3100 Pro-spectrophotometer (Amersham Biosciences, Piscataway, NJ) was used to measure the change in absorbance for all complexes. Background levels were measured in the absence of cell suspensions. All complex enzyme activities are expressed as nanomoles of substrate oxidized per mg^−^1 protein min^−^1 ml^−^1 (nmol/mg protein/min/ml).

#### Complex I (NADH-ubiquinone oxidoreductase)

Cell lysate (30–50 μg of protein/ml) was added to a cuvette containing 0.5 ml of reaction buffer (5 mM MgCl_2_, 2 mM KCN, 0.13 mM NADH, and 2 μg/ml Antimycin; diluted in K buffer). The reaction was started by the addition of Coenzyme Q_1_ (2 μl of 65 μM solution). At 180 seconds, 2 μl of Rotenone (2 μg/ml) was added to the cuvette. The change in absorbance at 340 nm, resulting from the oxidation of NADH, was measured using a kinetic program with 20 second intervals and a total of 18 readings.

#### Complex II (succinate-ubiquinone oxidoreductase)

Cell suspensions (30–50 μg of protein/ml) were added to a 1.5 ml microcentrifuge tube containing 0.5 ml of reaction solution 1 (5 mM MgCl_2_ and 20 mM succinate; diluted in K buffer), and then incubated in a 37°C water bath for 10 minutes. After transferring the cell solution to a cuvette, the reaction was started by the addition of 8 μl of reaction solution 2 (2 μg/ml Antimycin, 2 μg/ml Rotenone, 2 mM KCN, and 50 μM Dichlorophenlindophenol). At 180 seconds, 2 μl of Coenzyme Q_2_ (65 μM) was added to the cuvette. The change in absorbance at 600 nm, resulting from the reduction of Dichlorophenlindophenol, was measured using a kinetic program with 20 second intervals and a total of 18 readings.

#### Complex III (ubiquinol-cytochrome c oxidoreductase)

The reaction was started by the addition of 2 μl of Coenzyme Q_2_ (65 μM) to a cuvette containing 0.5 ml of reaction buffer (5 mM MgCl_2_, 2 mM KCN, 15 μM cytochrome c, 2 μg/ml of Rotenone, and 0.6 mM Dodecyl-β-d-maltoside; diluted in K buffer). At 60 seconds, a cell suspension (30–500 μg of protein/ml) was added to the cuvette. The change in absorbance at 550 nm, resulting from the reduction of cytochrome c, was measured using a kinetic program with 20 second intervals and a total of 12 readings.

#### Complex IV (cytochrome c oxidase)

Cell lysate (30 μg of protein/ml) was added to a cuvette containing 0.475 ml of assay buffer (10 mM Tris–HCl, pH 7.0, and 120 mM KCl), and the reaction volume was brought to 0.525 ml with enzyme dilution buffer (10 mM Tris–HCl, pH 7.0, and 250 mM sucrose). The reaction was started by the addition of 25 μl of ferrocytochrome c substrate solution (0.22 mM). The change in absorbance at 550 nm, resulting from the oxidation of cytochrome c, was measured using a kinetic program with a 5 second delay, 10 second intervals, and a total of 6 readings.

#### Citrate synthase

Cells in 100-mm dishes were washed twice with ice-cold PBS, and cells were harvested, centrifuged, and suspended in 100 μl of isolation buffer containing 1 M Tris, pH 7.4, and 3 M KCl. The reaction was started by the addition of cell lysate (10–30 μg of protein/ml) to a cuvette containing 150 μl of assay buffer (100 mM Tris, pH 7.4, 0.17 mM Oxaloacetic acid, 0.2 mM Acetyl CoA). The change in absorbance at 232 nm was measured using a kinetic program with 60 second intervals and a total of 8 readings.

#### TUNEL staining

The *In Situ* Cell Death Detection Kit, Fluorescein (Roche) was used as described. Cells (2 × 10^4^ cells/well) were grown in 8-well chamber slides until 70% confluent. Following incubation for 24 hours with 0.75 mM H_2_O_2_, the cells were fixed in 4% paraformaldehyde for 1 hour. Fixed cells were permeabilisated for 2 minutes on ice, followed by incubation with 75 μl TUNEL reaction mixture for 1 hour at 37°C. After washing twice with PBS followed by 5 minutes of nuclear staining with DAPI, the cells were imaged via confocal microscopy and the intensity of fluorescence (ex: 488 nm, em: 565 nm for TUNEL; ex: 358 nm, em: 461 nm for DAPI) was recorded to determine cells undergoing apoptotic cell death.

### Cyclophilin D studies

Immunoblotting, co-immunofluorescence, and co-immunoprecipitation assays were performed in the PC-12 altered cell lines at passages 1–8 to investigate the role of CypD.

#### Co-immunofluorescence staining

Cells (2 × 10^4^ cells/well) were grown in 8-well chamber slides until 70% confluent, and then fixed in 4% paraformaldehyde and 0.1% Triton X-100 for 30 minutes. Fixed cells were incubated with mouse anti-HSD10 (1:100, generated in our laboratory) and rabbit anti-CypD (1:200, generated in our laboratory), mouse anti-HSD10 (1:100) and rabbit anti-SODII (1:1000), or mouse anti-Hsp60 (1:1000) and rabbit anti-CypD (1:200) overnight, and then incubated with secondary antibodies (Alexa Fluor 488 anti-rabbit and Alexa Fluor 594 anti-mouse (1:2000, Invitrogen). DAPI was applied to the cells for 5 minutes followed by confocal microscopy. The intensity of fluorescence (ex: 499 nm, em: 520 nm for HSD10; ex: 343 nm, em: 442 nm for CypD; ex: 494 nm, em: 518 nm for SODII; ex: 495 nm, em: 519 nm for Hsp60; ex: 358 nm, em: 461 nm for DAPI) was recorded to determine HSD10 and CypD expression and localization to the mitochondrial markers, SODII and Hsp60.

#### Co-immunoprecipitation

Briefly, cells (10^6^ cells/dish) were grown in 150-mm dishes until fully confluent. Cells were washed twice with pre-chilled PBS, and then harvested, centrifuged, and suspended in 250 μl Co-Immunoprecipitation (Co-IP) buffer containing 150 mM NaCl, 50 mM Tris–HCl, pH 7.4, 1 mM EDTA, 0.5% NP-40, and 100X protease inhibitor (EMD Millipore). Cells were frozen and thawed in 250 μl Co-IP buffer for 10 cycles, followed by brief sonication and 30 minutes of lysis on ice. After centrifugation at 8000 × g for 5 minutes at 4°C, lysates were measured for protein concentration using the BCA protein assay and subjected to co-immunoprecipitation with antibodies. Samples with 500 μg protein extracts within 500 μl Co-IP buffer were incubated overnight with pull-down antibodies (rabbit anti-HSD10, generated in our laboratory; mouse anti-CypD, Abcam; rabbit IgG or mouse serum), while rotating at 4°C. The immunocomplexes were pulled out using Protein A/G-sepharose beads for 2 hr. Next, the immunoprecipitates were washed three times with Co-IP buffer, collected by brief centrifugation, and dissolved in denaturing sample buffer. Immunoblotting was used to reveal the immunoprecipitated proteins as previously described, and the antibodies used were mouse anti-CypD (1:8000), rabbit anti-HSD10 (1:3000), and mouse anti-Actin (1:8000).

### Animal studies

All animals were housed under pathogen-free conditions according to AAALAC guidelines. All animal-related experiments were performed in full compliance with institutional guidelines and approved by the Animal Care and Use Committee of the University of Kansas. Two-month old female severe combined immunodeficient (SCID) mice were purchased from Jackson Laboratory (Bar Harbor, ME).

Control and HSD10-overexpressing PC-12 cells were grown in 150-mm dishes until fully confluent. Cells were collected and inoculated into SCID mice via mammary fat pad injection after shaving of the area and alcohol preparation of the skin, using a sterile 22-gauge needle with 0.1 ml cell suspension of 1 × 10^6^ cells with manual restraint [26]. Mice were weighed and tumor size was measured using a caliper twice per week for a total of 32 days. Tumor volume was calculated using the formula: (length x width^2^)/2 [27]. Mice were imaged with an In-Vivo Multispectral FX PRO imaging system (Carestream, Woodbridge, CT) on day 30 of the experiment, 24 hours post-injection of 15 nmol of IRDye 800CW 2-deoxyglucose (2-DG, Li-Cor Biosciences, Lincoln, NB) optical probe [28] given via tail vein injection. An excitation filter of 760 nm and emission filter of 830 nm was applied for Near Infrared imaging to visualize tumor growth.

### Statistics

Paired t tests were used for statistical comparison of empty vector with HSD10 overexpression groups, and control shRNA with HSD10 shRNA groups. Unpaired t tests were used to analyze all animal data. All data was analyzed using StatView 5.0.1 Windows software (SAS Institute, Cary NC) and results are reported as mean ± SE. *P* < 0.05 was considered significant.

## Results

### Characterization of PC-12 HSD10-transfected cell lines

**Figure 1:**
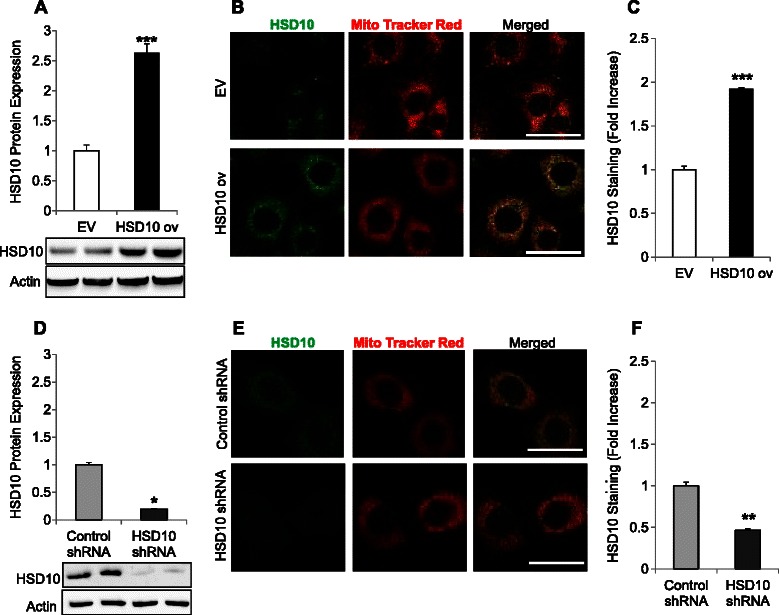
**Characterization of HSD10-transfected cell lines. A.** Empty vector (EV) and HSD10 overexpression (HSD10 ov) whole cell lysates were analyzed for HSD10 protein expression via immunoblotting. β-actin was used as the loading control, and HSD10 expression was normalized to actin (n = 4). **B.** Confocal microscopy demonstrating immunofluorescence staining of HSD10 alone (green), Mito Tracker Red alone (red), and these two antigens co-localized (yellow) in EV and HSD10 ov cells. Scale bar in **B**: 30 μm. **C.** Quantification of HSD10 immunofluorescence staining (depicted in **B**) displayed as fold increase (n = 5). **D.** Control shRNA and HSD10 shRNA whole cell lysates were analyzed for HSD10 protein expression via immunoblotting. β-actin was used as the loading control, and HSD10 expression was normalized to actin (n = 4). **E.** Confocal microscopy demonstrating immunofluorescence staining of HSD10 alone (green), Mito Tracker Red alone (red), and these two antigens co-localized (yellow) in control shRNA and HSD10 shRNA cells. Scale bar in **E**: 30 μm. **F.** Quantification of HSD10 immunofluorescence staining (depicted in **E**) displayed as fold increase (n = 5). Data presented as mean ± SE. *P < 0.01, **P < 0.001, ***P < 0.0001 verses control group.

After generating PC-12 cancer cell lines expressing either an empty vector (EV) or an HSD10 overexpression (HSD10 ov) vector, we first examined HSD10 protein expression levels in the cells using immunoblot analysis. HSD10 protein expression was increased by 3-fold in HSD10 ov cells compared to EV cells (Figure 1A). The level of HSD10 expression was further verified by immunofluorescence staining (Figure 1B). The intensity of HSD10 staining was significantly enhanced in HSD10 ov cells in comparison with EV cells (Figure 1C). In addition to confirming increased levels of HSD10 in the PC-12 overexpression cell lines, the immunofluorescence assay confirmed the localization of HSD10 to mitochondria as is depicted in the merged picture of Figure 1B.

To thoroughly examine the effect of HSD10 in cancer, we used lentiviral transfection to knockdown HSD10 in PC-12 cells. We again characterized the cells via immunoblot analysis, showing that HSD10 protein expression was significantly reduced by 80% in HSD10 shRNA-transfected cells in comparison with unrelated control shRNA-transfected cells (Figure 1D). The level of HSD10 knockdown was also confirmed by immunofluorescence staining of HSD10 (Figure 1E), which showed that the intensity of HSD10 staining was decreased in HSD10 shRNA cells compared to control shRNA cells (Figure 1F).

As HSD10 is typically located in mitochondria [12,29], specific localization likely promotes this enzyme’s multifunctional abilities within the cell. Therefore, our observation that HSD10 localizes to mitochondria in PC-12 HSD10-transfected cells suggests that fluctuating HSD10 levels may influence mitochondrial function. Thus, we next sought to examine the effect altered HSD10 expression has on mitochondrial bioenergetics.

### HSD10 overexpression enhances mitochondrial bioenergetics and membrane potential in PC-12 cells

**Figure 2:**
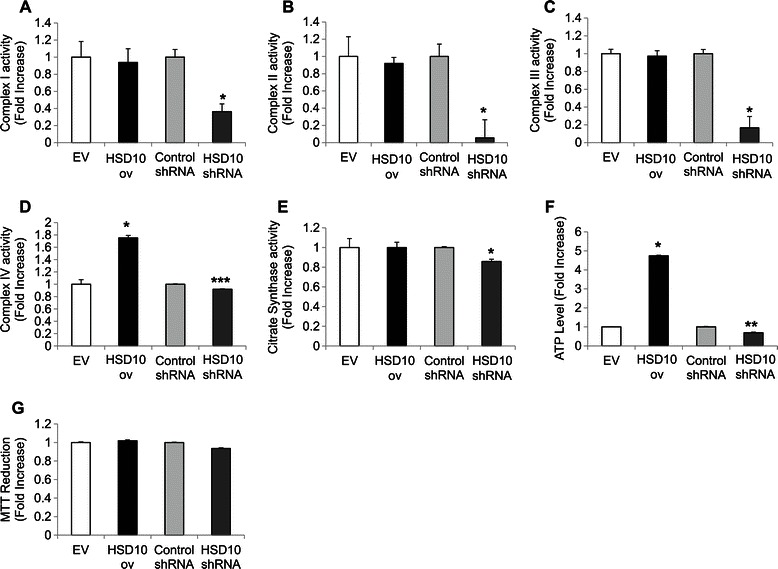
**Assessment of mitochondrial bioenergetics in HSD10-transfected cell lines.** Electron transport chain complex I **(A)**, II **(B)**, III **(C)**, and IV **(D)** enzyme activities were assessed in EV, HSD10 ov, control shRNA, and HSD10 shRNA cells. Results, displayed as fold increase (n = 5 for each assay), showed that complex IV activity is enhanced in HSD10 ov cells and all ETC. complex activities are decreased in HSD10 shRNA cells. **E.** Densitometry of citrate synthase enzyme activity (n = 5) showed no change in activity between EV and HSD10 ov cells, and reduced activity in HSD10 shRNA cells. **F.** Densitometry of ATP activity (n = 6) demonstrated that ATP levels are increased in HSD10 ov cells and diminished in HSD10 shRNA cells. **G.** Densitometry of MTT reduction (n = 4) exhibited similar reduction of MTT by all HSD10-transfected cell lines. Data presented as mean ± SE. *P < 0.01, **P < 0.001, ***P < 0.0001 versus EV and control shRNA groups.

To examine the effect of HSD10 on mitochondrial function, we assessed the enzyme activities of complexes I, II, III, and IV of the electron transport chain (ETC.). Complexes I, III, and IV are proton pumps, which generate the transmembrane proton gradient necessary to drive ATP generation by ATP synthase. Any changes in the ETC. system would impact mitochondrial ATP generation and any ensuing mitochondrial processes. While the enzyme activities of complexes I, II, and III remained unchanged (Figure 2A-C), complex IV activity was significantly increased in HSD10 ov cells compared with EV cells (Figure 2D), suggesting an enhancement in the ETC. system of HSD10-overexpressing cells. Conversely, HSD10 shRNA cells displayed significantly decreased ETC. complex enzyme activity in all of the complexes measured, in comparison to control shRNA cells (Figure 2A-D). This reduction in all of the complexes indicates that HSD10 is important for cancer cell functionality, and would likely have a substantial impact on subsequent mitochondrial processes.

Citrate synthase enzyme activity, which often serves as a quantitative enzyme marker for the presence of intact mitochondria, was similar between HSD10 ov cells and EV cells (Figure 2E). This indicated that, despite an overabundance of HSD10, the PC-12 cells were healthy. Next, we examined how the increase in complex IV activity impacted ATP production. In conjunction with the complex IV data observed in Figure 2D, the level of ATP was significantly elevated in HSD10 ov cells compared to EV cells (Figure 2F), demonstrating a possible increase in energy generation in HSD10-overexpressing PC-12 cells. On the other hand, citrate synthase enzyme activity, which serves as a measurement of mitochondrial fitness, was reduced in HSD10 shRNA cells (Figure 2E). This indicates that HSD10 knockdown disrupts mitochondrial function. Similarly, ATP production was diminished in HSD10 shRNA cells compared to control shRNA cells (Figure 2F), which was expected in view of the decreased activity observed in all of the ETC. complexes in Figure 2A-D.

As ATP is a key driver for many mitochondrial and cellular processes, we next examined the effect of HSD10 on cell viability by measuring cellular metabolic activity using the MTT reduction assay. MTT reduction levels remained similar between HSD10 ov cells and EV cells after 24 hours, indicating comparable cell viability (Figure 2G). Furthermore, the change in MTT reduction between control shRNA and HSD10 shRNA cells was not statistically significant after 24 hours (Figure 2G).

**Figure 3:**
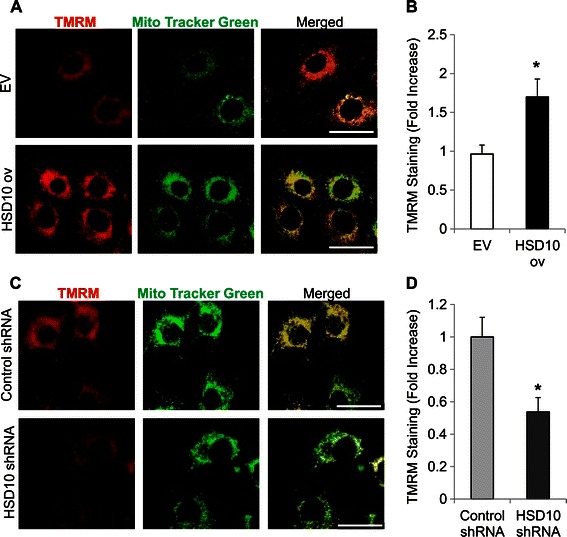
**Examination of mitochondrial membrane potential in HSD10-transfected cell lines. A.** Confocal microscopy demonstrating immunofluorescence staining of mitochondrial membrane potential with TMRM alone (red), Mito Tracker Green alone (green), and these two antigens co-localized (yellow) in EV and HSD10 ov cells. Scale bar in **A**: 30 μm. **B.** Quantification of TMRM immunofluorescence staining (depicted in **A**) displayed as the percentage of intensity of fluorescence (n = 4). **C.** Confocal microscopy demonstrating immunofluorescence staining of mitochondrial membrane potential with TMRM alone (red), Mito Tracker Green alone (green), and these two antigens co-localized (yellow) in control shRNA and HSD10 shRNA cells. Scale bar in **C**: 30 μm. **D.** Quantification of TMRM immunofluorescence staining (depicted in **C**) displayed as the percentage of intensity of fluorescence (n = 4). Data presented as mean ± SE. *P < 0.05 versus control group.

We further assessed mitochondrial function by examining the effect of HSD10 on mitochondrial membrane potential using the cell-permeant dye, TMRM. This red dye is readily taken up by active mitochondria and emits a stronger fluorescence in mitochondria with intact membranes [30], and co-localizes with Mito Tracker Green (Figure 3A). HSD10 ov cells displayed enhanced TMRM staining as opposed to the normal levels observed in EV cells (Figure 3A-B). Alternatively, HSD10 shRNA cells displayed a decrease in TMRM staining intensity compared to control shRNA cells (Figure 3C-D). Due to the significant increase in TMRM fluorescence observed in HSD10 ov cells, we propose that HSD10 overexpression promotes mitochondrial membrane hyperpolarization. Whereas loss of mitochondrial membrane potential can induce cell death pathways, we suggest that hyperpolarization mediated in part by HSD10 would lead to protection against induction of cell death. In HSD10 shRNA cells, there is considerably less HSD10 present within mitochondria; hence we theorize that HSD10 knockdown would induce mitochondrial membrane depolarization, thus increasing the chance of cell death induction.

### HSD10 overexpression increases PC-12 cell growth in cell culture and *in vivo*

**Figure 4:**
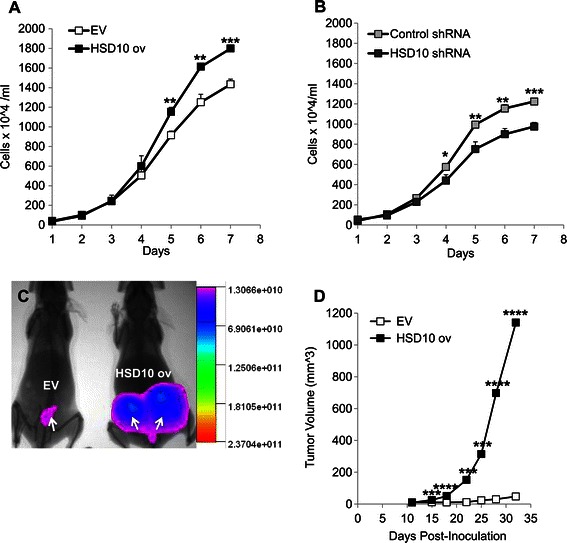
**Effect of HSD10-modification on*in vitro*cell growth and*in vivo*tumor growth. A.** Growth curve depicting the number of EV and HSD10 ov cells measured over seven days demonstrated that HSD10 ov cells grew faster in cell culture. Results displayed as cells × 10^4^ per ml (n = 9). **B.** Growth curve displaying the number of control shRNA and HSD10 shRNA cells measured over seven days showed that HSD10 shRNA cells grew slower than control shRNA cells. Results depicted as cells × 10^4^ per ml (n = 9). **C.** EV and HSD10 ov cells were injected into the mammary fat pad tissue of 20 two-month old female SCID mice. Day 30 tumor growth in two SCID mice inoculated with EV (left mouse) or HSD10 ov (right mouse) cells demonstrated considerable tumor growth in the HSD10 tumor mouse when observed beside the EV tumor mouse. Visualization of tumors was performed 24 hours post-injection of 15 nmol 2-DG optical dye with an In-Vivo Multispectral FX PRO imager. Arrows point to tumors. **D.** Quantification of tumor growth in all SCID mice injected with EV (n = 8) or HSD10 ov (n = 12) cells grown over a total of 32 days, depicted in tumor volume (mm^3^). Data presented as mean ± SE. *P < 0.05, **P < 0.01, ***P < 0.001, ****P < 0.0001 versus control group.

After evaluating the effect of HSD10 on mitochondrial processes, we determined the effect of HSD10 overexpression on cancer cell growth. Using *in vitro* cell culture, we performed growth rate curves using both PC-12 HSD10 overexpression and PC-12 HSD10 knockdown cell lines. As shown in Figure 4A, HSD10 ov cells grew at a significantly faster rate compared to EV cells over seven days. Knockdown of HSD10 led to a considerable decrease in growth rate compared with control shRNA cells (Figure 4B). Taken together, these results suggest that HSD10 promotes pheochromocytoma cell growth in cell culture and that knockdown of HSD10 has a reverse effect on cancer cell growth.

Xenograft tumor models were used to validate the role of HSD10 on cancer cell growth *in vivo*. SCID mice inoculated with HSD10 ov cells exhibited drastically larger tumors compared to mice with EV tumor xenografts which displayed very minimal growth (Figure 4C-D). These results demonstrate that HSD10 overexpression accelerates tumor development *in vivo*, providing further evidence that HSD10 is an important tumorigenic mediator in the transformation of non-malignant adrenal gland cancer.

### HSD10 overexpression enhances PC-12 cell resistance to oxidative stress stimuli

**Figure 5:**
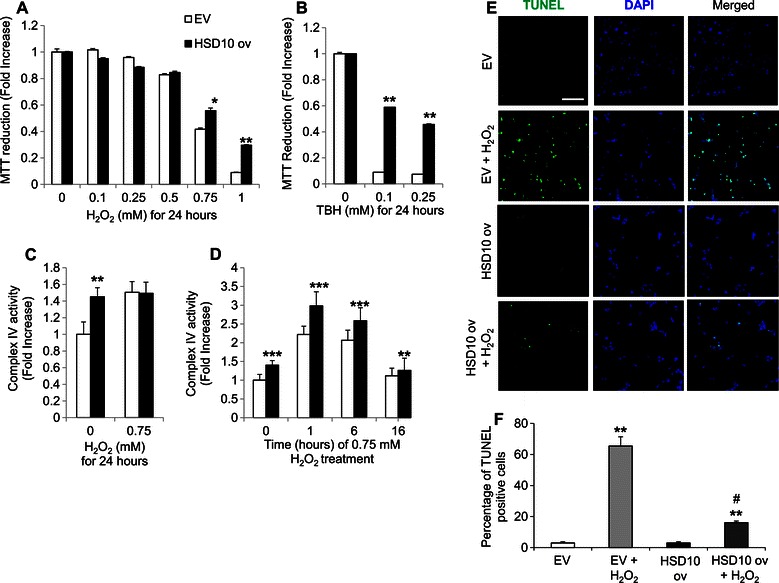
**Effect of HSD10 overexpression on cellular resistance to death-inducing stimuli. A-B.** Densitometry of MTT reduction in EV and HSD10 ov cells treated with **A)** 0, 0.1, 0.25, 0.5, 0.75, and 1 mM H_2_O_2_ (n = 3 for all groups), and **B)** 0, 0.1, and 0.25 mM TBH for 24 hours (n = 3 for all groups). Results demonstrated that HSD10 ov cells were more resistant to oxidative stress-induced cell death. **C-D.** ETC. complex IV enzyme activity was assessed in EV and HSD10 ov cells treated with 0.75 mM H_2_O_2_ for **C)** 24 hours (n = 3), and **D)** 0, 1, 6, and 16 hours (n = 6 for all time points). Results, displayed as nmol/mg protein/min/ml, showed that complex IV activity is enhanced in HSD10 ov cells under an oxidative stress condition. **E.** Confocal microscopy demonstrating TUNEL staining of cells undergoing apoptosis (green), nuclear staining with DAPI (blue), and these two antigens co-localized (merged) in EV and HSD10 ov cells, treated with 0 mM and 0.75 mM H_2_O_2_ for 24 hours. Scale bar in **E**: 30 μm. **F**. Quantification of TUNEL staining (depicted in **E)** displayed as the percentage of TUNEL positive cells (n = 4). Data presented as mean ± SE. *P < 0.05, **P < 0.01, ***P < 0.0001 versus EV control group (A-D) and versus EV and HSD10 ov non-treatment groups **(F)**; #P < 0.01 versus EV treatment group **(F)**.

To assess resistance to cell death, we treated the PC-12 HSD10 overexpression cells with various concentrations of H_2_O_2_ and tert-butyl hydroperoxide (TBH) for 24 hours to stimulate oxidative stress conditions as cancer cells are typically exposed to higher oxidative stress levels [31]. While cell viability steadily decreased for both cell groups as the chemical dosage increased, HSD10 ov cells demonstrated significantly higher reduction of MTT at 0.75 and 1 mM concentrations of H_2_O_2_ compared to EV cells (Figure 5A). Treatment of the cells with TBH showed similar results, with HSD10 ov cells reducing considerably more MTT compared to EV cells at the lowest dosage of TBH given (0.1 mM, Figure 5B). Thus, while these two oxidative stressors reduce cell viability in both EV and HSD10 ov cells, PC-12 cells with HSD10 overexpression exhibited more resistance to chemical-induced oxidative stress.

We tested this cellular resistance further by examining complex IV enzyme activity. We chose to treat the cells with 0.75 mM of H_2_O_2_ as the amount of MTT reduced by EV cells remained at a higher level compared to the amount seen at 1 mM H_2_O_2_ (Figure 5A) and the lowest dose of TBH used (Figure 5B). As a starting point, we assessed complex IV activity in the PC-12 HSD10-overexpressing cells treated with 0.75 mM of H_2_O_2_ for 24 hours. However, H_2_O_2_ treatment showed no difference in enzyme activity between the two groups (Figure 5C). Rationalizing that any changes in enzymatic activity would likely be more visible earlier in the chemical treatment, we assessed the activity of complex IV at several time points during the 24 hour period. As speculated, complex IV enzyme activity was significantly increased in HSD10 ov cells compared to EV cells after just one hour of H_2_O_2_ treatment (Figure 5D). Indeed, this difference in activity occurred earlier into the treatment period as the difference between HSD10 ov and EV cells at 16 hours of H_2_O_2_ treatment, while still statistically different, returned to similar levels (Figure 5D). This data implies that HSD10 overexpression aids cell survival under oxidative stress settings, conceivably by elevating and/or maintaining mitochondrial bioenergetics, such as ETC. activity and ATP generation, during this death-inducing condition.

To provide further evidence for this concept, we performed TUNEL staining in EV and HSD10 ov cells without treatment, and EV and HSD10 ov cells treated with 0.75 mM H_2_O_2_ for 24 hours (Figure 5E). As expected, both EV and HSD10 ov cells treated with H_2_O_2_ exhibited higher percentages of cells undergoing apoptosis (Figure 5F), compared with the untreated matched control groups. However, the HSD10 ov treatment group had significantly less TUNEL staining compared to the EV treatment group (Figure 5F, #p-value), indicating that cells overexpressing HSD10 are more protected from apoptosis induction. The data presented here support the concept that HSD10 overexpression increases pheochromocytoma cell resistance to cell death induced by oxidative stress.

### HSD10 overexpression increases HSD10-CypD complex formation in PC-12 cells

**Figure 6:**
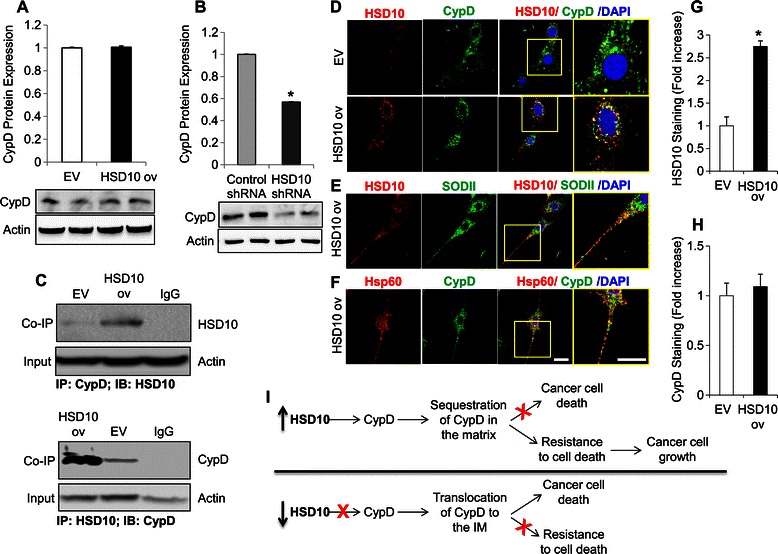
**Effect of HSD10-modification on CypD and how it may influence cancer cell growth and death. A.** EV and HSD10 ov whole cell lysates were analyzed for CypD protein expression using immunoblotting. β-actin was used as the loading control, and CypD was normalized to actin (n = 4). **B.** Control shRNA and HSD10 shRNA whole cell lysates were analyzed for CypD protein expression using immunoblotting. β-actin was used as the loading control, and CypD was normalized to actin (n = 4). **C.** EV and HSD10 ov whole cell lysates were analyzed for HSD10-CypD complexes using co-immunoprecipitation. β-actin was used as the loading control for the input. The immunoblots demonstrate an increased HSD10-CypD interaction in PC-12 cells overexpressing HSD10 compared to EV cells. **D.** Confocal microscopy demonstrating immunofluorescence staining of HSD10 alone (red), CypD alone (green), and these two antigens co-localized (yellow) in EV and HSD10 ov cells. **E.** Immunofluorescence staining of HSD10 alone (red), mitochondrial marker SODII alone (green), and these two antigens co-localized (yellow) in HSD10 ov cells. **F.** Immunofluorescence staining of CypD alone (green), mitochondrial marker Hsp60 alone (red), and these two antigens co-localized (yellow) in HSD10 ov cells. Scale bar in **F**: 20 μm. **G-H.** Quantification of HSD10 and CypD fluorescence densities (depicted in **D**) displayed as fold increase (n = 4). Data presented as mean ± SE. *P < 0.01 versus transfected control group. **I.** Hypothetical mechanism of action for HSD10-mediated cancer cell growth. Top panel, cells overexpressing HSD10 bind to CypD and sequester it in the mitochondrial matrix, thereby avoiding cell death induction; this resistance allows for continued cancer cell proliferation. Bottom panel, cells under-expressing HSD10 cannot bind all of the available CypD; thus unbound CypD translocates to the IM where it induces cell death via MPTP opening.

Next, we sought to determine the mechanism behind the ability of HSD10 to regulate cancer cell growth and cell death resistance associated with mitochondrial function. We examined the relationship between HSD10 and CypD, which is a modulator of MPTP-opening and cell death induction under stress conditions. We postulate that HSD10 aids in cancer cell resistance by preventing MPTP-induced cell death via enhanced binding to CypD. Using immunoblot analysis, we determined that CypD protein expression remains similar between HSD10 ov cells and EV cells (Figure 6A). This result is consistent with other studies of CypD in cancer [32]. Interestingly, CypD protein expression was significantly reduced in HSD10 shRNA cells compared with control shRNA cells (Figure 6B). This indicates that while CypD expression remains level during HSD10 overexpression, it is negatively impacted by HSD10 reduction. We suggest that, due to the reductions in both HSD10 and CypD, cancer cells become more susceptible to cell death induction.

Additionally, we performed co-immunoprecipitation assays to investigate HSD10-CypD interactions in the PC-12 overexpression cell lines. As shown in Figure 6C, there is an enhanced interaction between HSD10 and CypD in HSD10 ov cells compared to EV cells, which was confirmed using both proteins as pull-down antibodies. This indicates that increased levels of HSD10 promote the formation of HSD10-CypD complexes. The co-localization of HSD10 and CypD within mitochondria was further confirmed via immunofluorescence (Figure 6D). The expression pattern of CypD did not change between EV and HSD10 ov cells (Figure 6H), which is consistent with the CypD protein expression data shown in Figure 6A. As expected, HSD10 levels where increased in HSD10 ov cells compared to EV cells (Figure 6G), consistent with the results in Figure 1A-C. Co-staining of HSD10 and CypD with the mitochondrial markers SODII and Hsp60, respectively, confirms mitochondrial localization of the proteins (Figure 6E-F). Although CypD expression remains constant between EV and HSD10 ov cells, the increased expression of HSD10 in HSD10 ov cells appears to correspond with enhanced co-localization of HSD10 and CypD as shown in the merged images of Figure 6D. Taken together, the results imply that an increased abundance of HSD10 in the vicinity of CypD leads to HSD10-CypD complex formation in PC-12 cells, conceivably preventing cell death induction. These findings further solidify the concept that HSD10 is important in promoting cancer development and maintenance.

## Discussion

We have demonstrated for the first time that PC-12 pheochromocytoma cells overexpressing HSD10 grow significantly faster in cell culture and form larger tumors at a faster rate in SCID mice. We theorize that HSD10 promotes enhanced cell growth through altered mitochondrial function, as we observed enhanced complex IV enzyme activity and ATP production in PC-12 cells overexpressing HSD10. Knockdown of HSD10 negatively impacted PC-12 cell growth and mitochondrial function. All ETC. complex activities were considerably reduced, resulting in decreased ATP generation. This diminished energy production is likely responsible for the reduced growth rate observed in cell culture. We also evaluated the possibility that HSD10 may confer protection in cancer cells. Upregulation of HSD10 permitted PC-12 cells to maintain a higher functional capacity with reduced cell death induction under chemical-induced oxidative stress situations. Taken together, these findings provide evidence that HSD10 mediates cancer cell growth and resistance to death-inducing environments.

Upregulation of MPTP components correlates with increased cancer cell proliferation and resistance to MPTP-induced cell death [33,34]. Therefore, in order to uncover the mechanism underlying HSD10-mediated cancer cell growth and resistance, we determined whether interactions existed between HSD10 and MPTP components, such as the MPTP regulatory component, CypD. During environmental stress, CypD translocates from the matrix to the inner mitochondrial membrane (IMM) where it then initiates the opening of the MPTP, consequently leading to cell death [35]. Suppression of MPTP-induced cell death observed in tumor cells is thought to occur due to CypD molecular interactions which prevent IMM translocation, as inhibition of CypD protects malignant cells from necrosis [36]. During tumorigenesis, overexpression of CypD inhibits cell death induction in tumor cells due to interactions with cell death proteins, such as anti-apoptotic Bcl-2 [37] and hexokinase II [38].

In our studies, CypD expression remained the same in PC-12 HSD10 overexpression cell lines; however, CypD expression was greatly reduced in HSD10 shRNA cells. Furthermore, HSD10 and CypD exhibited enhanced co-localization within mitochondria and increased formation of HSD10-CypD complexes in PC-12 cells overexpressing HSD10. Applying this data together, Figure 6I demonstrates our model for the mechanism of action for HSD10-mediated cancer cell growth and resistance to cell death. As shown on the upper half of the figure, cancer cells overexpressing HSD10 have reduced MPTP-mediated cell death due to enhanced interactions between HSD10 and CypD thereby preventing CypD translocation to the IMM. As depicted on the lower half of Figure 6I, cancer cells with reduced HSD10 levels are more vulnerable to MPTP-induced cell death as fewer molecules of HSD10 are able to bind and retain CypD in the matrix.

In addition to the HSD10-CypD interaction, this study identified other interesting pathways. Typically, tumors exhibit increased cell proliferation and resistance to cell death. Similarly, HSD10-overexpressing PC-12 cells demonstrated an enhanced cell growth rate and resistance to cell death induced by oxidative stressors. This phenomenon is likely due to altered processes within mitochondria, as large amounts of HSD10 localized with mitochondria in HSD10-overexpressing cells. Although the Warburg effect of elevated glycolytic ATP production in cancer cells is widely recognized [39], many groups have revealed that mitochondria in tumor cells are able to operate both respiratory pathways [40]. As we observed elevated complex IV activity in HSD10-overexpressing cells, we postulate that HSD10 provides additional energy metabolites for the cells.

Among its many functions, HSD10 metabolizes β-hydroxybutyrate (BHB), which can be used as an energy source when blood glucose levels are low [41]. Studies have shown that tumor animal models utilize BHB as an energy source. For instance, it has been observed that BHB concentrations increased in the tumors of nude rats as tumor blood flow decreased [42]. Additionally, it was recently discovered that BHB is upregulated in caveolin-1 null mice and proposed that epithelial cancer cells directly take in stromal-derived BHB to drive tumor progression and eventual metastasis [43]. With cancer cells already displaying heightened ATP production due to the two principal energy metabolism pathways, the addition of another source of energy via the enzymatic capability of HSD10 would increase ATP generation even further. This could lead not only to alterations in mitochondrial bioenergetics, but also to changes in mitochondrial signal transduction pathways, particularly resistance to cell death.

Most often, cancer cells have disrupted cell death pathways due to mutations that either inhibit pro-apoptotic proteins [44] or elevate anti-apoptotic proteins [45]. As depicted by the model in Figure 6I, the suppression of MPTP-mediated apoptosis is due to the sequestration of CypD by HSD10; our future efforts will focus on this pathway. Additionally, CypD has been shown to interact with anti-apoptotic proteins, including Bcl-2. It is possible HSD10 may bind to CypD and displace Bcl-2, allowing Bcl-2 to prevent apoptosis. Validation of the role of Bcl-2 in this mechanism will require additional studies. Also, as HSD10 is a versatile enzyme within cells, further inquiry into its potential role in the repression of oxidative stress-induced cell death would be an interesting undertaking.

Lastly, using an *in vivo* xenograft mouse model, we showed that HSD10-overexpressing cells grew faster and larger over 32 days as opposed to vector control tumors. While there are limited *in vivo* xenograft studies involving PC-12 cells, one group revealed that following implantation of PC-12 cells into the striatum of Sprague–Dawley rats, the number of cells remained unchanged without continued tumor growth [46]. This study supports the minimal growth observed in our EV tumors and provides further support for the tumorigenic ability of HSD10 (Figure 4C-D).

The data presented here provides a promising platform for further research to elucidate the mechanism underlying HSD10-mediated cancer cell growth and cell death resistance. Our laboratory recently created small molecule inhibitors of HSD10 [47]. As HSD10 overexpression grants pheochromocytoma cells enhanced cellular proliferative and cell death resistant capabilities, targeted inhibition of HSD10 in cancer cells may provide a novel treatment method. Furthermore, now that we have verified that HSD10 is important in adrenal gland tumor cell development, we intend to investigate its role in other human cancers. The effect of HSD10 in breast cancer development will be especially compelling as HSD10 is able to regulate estrogen steroidogenesis [10]. Furthermore, fellow HSD10 family member HSD17B type 1 was discovered as a novel target for endocrine therapy in certain breast cancer patients [48]. Evaluating the effect of HSD10 on human cancers would then provide more information as to its translational implications as a biomarker or treatment target.

## Conclusions

In summary, we have provided substantial evidence demonstrating that HSD10 overexpression significantly increased pheochromocytoma cell growth in cell culture and *in vivo*. The increases in respiratory enzymes and energy generation observed in HSD10-overexpressing cells likely supported the accelerated growth rate. Furthermore, HSD10 upregulation provided added protection against oxidative stress stimuli. These findings indicate that HSD10 is involved in cancer cell growth and development. This study shows that HSD10 provides a promising novel target for cancer therapy.

## Abbreviations

HSD10: 17β-hydroxysteroid dehydrogenase type 10
TBH: Tert-butyl hydroperoxide
K buffer: Potassium buffer
EV: Empty vector
HSD10 ov: HSD10 overexpression
